# Applying refinement to the use of mice and rats in rheumatoid arthritis research

**DOI:** 10.1007/s10787-015-0241-4

**Published:** 2015-07-14

**Authors:** Penny Hawkins, Rachel Armstrong, Tania Boden, Paul Garside, Katherine Knight, Elliot Lilley, Michael Seed, Michael Wilkinson, Richard O. Williams

**Affiliations:** Research Animals Department, RSPCA, Wilberforce Way, Southwater, West Sussex RH13 9RS UK; Huntingdon Research Centre, Woolley Road, Alconbury, Cambridgeshire PE28 4HS UK; UCB Celltech, 208, Bath Road, Slough, SL1 3WE UK; Institute of Infection, Immunity and Inflammation, College of Medical, Veterinary and Life Science, University of Glasgow, Sir Graeme Davies Building, 120, University Place, Glasgow, G12 8TA Scotland, UK; Home Office Animals in Science Regulation Unit, 2, Marsham Street, London, SW1P 4DF UK; Medicines Research Group, University of East London, Romford Road, London, E15 4LZ UK; Biological Services, Veterinary Research Facility, University of Glasgow, 464, Bearsden Road, Glasgow, G61 1QH Scotland, UK; Kennedy Institute of Rheumatology, University of Oxford, Roosevelt Drive, Headington, Oxford, OX4 7FY UK

**Keywords:** Rheumatoid arthritis, Animal model, Refinement, Three Rs, Ethical review, Humane endpoint, Welfare assessment, Experimental design, Autoimmune disease

## Abstract

Rheumatoid arthritis (RA) is a painful, chronic disorder and there is currently an unmet need for effective therapies that will benefit a wide range of patients. The research and development process for therapies and treatments currently involves in vivo studies, which have the potential to cause discomfort, pain or distress. This Working Group report focuses on identifying causes of suffering within commonly used mouse and rat ‘models’ of RA, describing practical refinements to help reduce suffering and improve welfare without compromising the scientific objectives. The report also discusses other, relevant topics including identifying and minimising sources of variation within in vivo RA studies, the potential to provide pain relief including analgesia, welfare assessment, humane endpoints, reporting standards and the potential to replace animals in RA research.

## Introduction

Rheumatoid arthritis (RA) is a painful, chronic autoimmune disorder. Current treatments include non-steroidal anti-inflammatory drugs (NSAIDs), disease-modifying anti-rheumatic drugs (DMARDs) and biologics, but none are curative and there is a significant ‘non-responder’ rate (Strand et al. 2007; Julià et al. 2009). There is thus a need to develop more effective treatments for RA. In vivo studies, using animal ‘models’ of RA, are currently part of the research and development process for new or improved therapies and treatments.

However, procedures used to induce arthritis in animals can cause suffering, which may be mild, moderate or severe, depending upon the model and the duration of the study. Implementation of the three Rs (replacement, reduction and refinement) is thus a priority.

This document complements the literature on good practice within RA research by providing practical information on refinement that is often not included in publications. It is intended for a wide audience; researchers, animal technologists, ethics or animal care and use committees, veterinarians, funding bodies, regulators and anyone designing or reviewing studies involving in vivo models of arthritis worldwide.

## Selecting the most appropriate approach and ‘model’

As a starting point, it is clearly essential to fully consider the mechanistic applicability and translatability of the various in silico, in vitro, in vivo and clinical methodologies used to address scientific questions relating to RA. Replacing or avoiding animal use should be a principal goal, as required in many legislations including European Directive 2010/63/EU (European Commission 2010).

For example, developments in three-dimensional tissue modelling show promise for in vitro drug evaluation, including in anti-inflammation research (Peck and Wang 2013), and in vitro cultures of bovine and human chondrocytes and cartilage discs are already used to investigate mechanisms of cartilage destruction in RA (Neidhart et al. 2000; Pretzel et al. 2009). Synoviocyte models using cells obtained from human RA or osteoarthritis patients during surgery have also been developed as test systems for candidate therapeutics (Smolian et al. 2001; Ribel-Madsen et al. 2012). It is important that use of human material is maximised—especially as fewer replacement surgeries are conducted in RA because patients are treated more successfully with DMARDs and biologics. Biologics with specific and well-defined molecular targets have also led to opportunities to replace animal RA models; e.g., analysis of immune and inflammatory parameters in peripheral blood mononuclear cells from patients before and after biologic therapy can reduce the need for animal use in some cases.

It is good practice to keep up with progress in such techniques, which can help to replace or avoid animal use, for example by screening out compounds without therapeutic benefit. Databases of in vitro, epidemiological and in silico models can help with this, e.g., http://www.go3r.org.

If there is no scientifically viable alternative approach, the choice of animal model should be guided by animal welfare considerations, aiming to minimise suffering in addition to the scientific purpose.^1^ There are useful reviews of animal models of RA in the literature (Bevaart et al. 2010; Patel et al. 2010; Bolon et al. 2011; Kollias et al. 2011; Vincent et al. 2012). Readers are also referred to initiatives such as ‘Be The Cure’ (BTCURE), a pan-European research program to develop new RA therapies (www.btcure.eu). One of its aims is to develop an infrastructure to standardise procedures for generating and interpreting commonly used RA animal models.

These can be broadly divided into those that are (i) spontaneous, including mutant and genetically altered strains, and (ii) induced. Spontaneous models progress naturally and generally involve a non-resolving, chronic condition. Induced models may be polyarthritic (involving a systemic response), which are more likely to be severe, or monoarthritic (induced by local challenge into a joint).

Species used in RA studies worldwide include non-human primates (marmosets and macaques), mice, rats, rabbits, zebrafish, pigs and dogs, but this report addresses mice and rats as they are most commonly used.

### Scientific issues

There is some debate about the optimum in vivo RA models for particular disease aspects, as well as their translatability to human disease, with different models possessing different mechanistic and clinical features (Vincent et al. 2012). If animal models fail, it may be with respect to clinical predictivity, or to misapplication of the type of validity required, be this ‘face validity’ (similarity to the human disease features of interest), ‘construct validity’ (similar underlying biological mechanisms) or ‘predictive validity’ (whether there is a similar response to clinically effective therapeutic agents) (McGonigle and Ruggeri 2014). For further explanation and discussion of causes of reduced external validity, see van der Worp et al. (2010). A ‘pathogenesis map’ for RA to assist with the decision-making process regarding validity is set out in Vincent et al. (2012).

### Ethical and animal welfare issues

A ‘harm-benefit assessment’, in which the potential harms to animals (i.e., pain, suffering or distress) are considered against the possible benefits of each project, is commonly used by regulators and ethics committees to make decisions about the justification for animal use (e.g., European Commission 2010; National Research Council 2011). The project should also have realistic objectives that are deliverable in practice.

With respect to identifying harms, factors to consider include:

- whether arthritis is spontaneous or an inducer is necessary;
- any need to boost or synchronise;
- number and frequency of interventions including anaesthesia;
- latency to onset of severe outcomes and their subsequent duration;
- number of joints affected;
- maximum level of suffering experienced by the animal;
- lifetime suffering experienced by the animal;
- potential to provide analgesia;
- numbers of animals required.

The Group considered the welfare impact of poly- and monoarthritic models, with respect to severity and the number of joints affected. The consensus was that some of the classical polyarthritic models (e.g., adjuvant- or collagen-induced models) generally result in a more severe arthritis and so should only be used as translational tools if there is strong supporting evidence of a relevant disease mechanism. Low severity (as opposed to hyperalgesia) monoarthritic models, including zymosan-induced and antigen-induced arthritis (Patel et al. 2010; Vincent et al. 2012), possess many relevant disease processes and, being less severe, should be used instead of polyarthritic models wherever possible. In addition, if only one limb is affected then the animal is able to compensate by redistributing weight between the other three limbs.

Harm-benefit assessments may be more complex for novel models that are less well established. Some may be more severe, e.g., the SKG mouse which develops severe arthritis plus extra-articular inflammation (Yoshitomi et al. 2005), while others can be manipulated to have reduced severity with greater clinical relevance (e.g., the KBxN serum transfer model; Montero-Melendez et al. 2011).

### Sources of variation in disease development and progression

A number of factors can lead to variations in the incidence, severity and timing of arthritis, which can affect the number of animals needed to obtain a statistically significant result. Potential sources of variation, and actions that can be taken to minimise these, are set out in Table 1.

**Table 1:** Sources of variation in animal models of RA

Sources of variation	Ways of addressing these
Protocol	
Variation in protocols for inducing arthritis	Search the literature for suitable standardised protocols^a^ and consult colleagues Monitor progress with initiatives such as BTCURE
Different protocols for assessing outcomes, e.g., with respect to clinical assessment, welfare assessment or histopathology	Research accepted assessment methods, ensuring discriminative power (see below) Adapt the histopathology score to the specific model
Different protocols for assessing therapy efficacy, given that the relevance of the drug target depends on the underlying mechanism	Research the appropriate efficacy measure for each target and define outcome-based assessment criteria for potential therapies, e.g., clinical scores or cellular responses
Environmental disturbance due to husbandry, scientific procedures and observations, or maintenance/construction work	Keep noise to a minimum Capture and handle animals with care Minimise the number of technical acts (e.g., administration of substances and anaesthesia)—but not by increasing the impact on the animal (e.g., without excessive dose volumes) Reduce husbandry and maintenance procedures, like cage cleaning and cleaning animal rooms, to the minimum necessary for good health Find out when cages and rooms are cleaned and avoid conducting procedures immediately afterwards
Statistical power	Conduct power calculations, use appropriate numbers, and define appropriate statistical analysis at the project planning stage. Plan for ‘dropouts’ Use pilot experiments, where appropriate, to define acceptable limits of severity and ensure statistical power
Variation in batches and quality control of biologicals such as collagen, lipopolysaccharide and *Mycobacterium*	Only use defined and/or batch tested biologicals, ensuring they are in date
Animals	
Different species, strains, sexes or ages of animals NB Some outbred strains are cheaper than inbred and can respond with arthritis—but due to genetic variability, severity can range from no response to extreme	Search the literature to help select the appropriate animal—but be critical and do not simply follow tradition; research and review current approaches with respect to species, sex, strain and age Use inbred strains to reduce variability and extremes in responses
Lack of proper colony management, leading to (i) genetic contamination (ii) incomplete inbreeding or (iii) genetic drift, resulting in unpredictable variations in susceptibility	Ensure good colony management; ‘refresh’ in-house colonies periodically by returning to founder stock; ensure frequent genotyping of generations Consider establishing a colony for long term projects, but ensure overbreeding and wastage are minimised
Variations in health status, e.g., pinworm infection	Apply good health care and colony management, led by animal technologists and the attending veterinarian
Environment	
The length of time that animals have spent in the facility; i.e., animals housed for longer before induction may be more susceptible to arthritis in the case of collagen models	See comments for ‘variation in protocols for inducing arthritis’ above
Level of biocontainment, i.e., whether in individually ventilated cages (IVCs) or conventional microbial environment	Be aware of model-specific pros and cons, e.g., mouse C57Bl6 CAIA seems to require IVC housing, but the SCW model may be more responsive in an open-top cage environment
Type of litter, nesting material, enrichment items and diet; interactions with humans	Ensure that these are carefully selected and adequately described in publications (see Sect. 3)
Operator effects	
Variations in performance of techniques, with respect to expertise or level of awareness of correct protocol—an establishment ‘culture’ issue	Ensure that good practice is observed with training, supervision, assessment of competence and Continuing Professional Development, seeking advice both internally and externally as necessary Account for any variation in experimental design; block by operators (personnel conducting each procedure)
Other sources of variation	
Unexplained variation between different facilities using the same protocols, sexes and strains	Ensure good attention to detail at all steps when inducing arthritis Liaise with external colleagues and compare how protocols are interpreted Discuss the animals’ experiences with animal technologists and care staff to identify differences in husbandry protocols Monitor and compare physical environments, e.g., noise, temperature fluctuations Conduct a full health screen of animals Ensure that publications include an appropriate level of detail to help interpret results if differences persist

^a^Standardised protocols can help to promote consistency, but should be critically considered every time. ‘Standardised’ does not always mean ‘fit for purpose’, and standard protocols may involve greater animal numbers or suffering than is desirable. An alternative approach to standardisation between facilities is for each to use models that enable good reproducibility with minimal suffering, and regular refinement, ensuring that protocols are written up in adequate detail. This approach also further disseminates information about good practice

## Refinement of animal models

Once the most appropriate model has been chosen, the next step is to ensure that refinement is fully implemented. An effective approach is to set out the whole life experience of the animal and consider how each potentially painful or distressing event could be refined, collectively leading to a significant overall reduction in suffering (European Commission 2012).

Table 2 sets out possible adverse effects within RA studies, with potential ways of ameliorating these. Supplementary text to explain some of the entries is set out below.

**Table 2:** Adverse effects and refinement

Potential adverse effect	How this may be refined
Administration of RA inducer	
Capture, handling and restraint	Competent, empathetic capture and handling (e.g., capture by cupping or tunnel, not tail) Habituation to handling and restraint
Pain due to administration of inducer (intradermal or subcutaneous injection)	Use gaseous anaesthesia for intradermal routes, to reduce pain and increase accuracy Inject intradermally into the rump, not the tail base. If the site is painful, capture or restraint by the tail will hurt Keep volumes and doses to the minimum necessary; it is better to use multiple sites if larger volumes are needed—but not too close together as injectates can coalesce, causing granulomas Ensure injectate has been formulated so as to minimise swelling and pain
After effects of anaesthesia e.g., dehydration, inappetence	Give treats such as Nutella^®^ or sunflower seeds Ensure animals can reach water or food with high water content such as wet mash, transgel, satsuma segments Provide oral glucose or rehydration with saline if necessary Monitor body mass and dehydration
Pain or ulceration around injection site	Inject into the rump for less risk of ulceration; additional injections can be into the flank if needed Never inject into the foot—this is too painful and not necessary If animal pays attention to injection site, apply topical local anaesthetic and review anaesthesia and injection protocol Ulceration should heal after 4 to 7 days—if not, implement a humane endpoint based on characteristics and persistence (see Sect. 7) Consider needle gauge with care and avoid ‘tracking’; implement humane endpoint if significant tracking
Administration of inducer by intraperitoneal injection	This is ‘going blind’ and adequate training is essential in order to avoid injecting into an organ or the gut. Never administer Freund’s complete adjuvant (FCA) via this route
Specific adverse effects due to adjuvant, e.g., granuloma, irritation, lesions	Use the least harmful adjuvant possible—monitor the literature for alternatives and challenge the use of problematic compounds like FCA Shaving while under anaesthesia, immediately before injection, helps to monitor adverse effects if these are likely Trial using incomplete Freund’s adjuvant for a less severe reaction
Effects of lipopolysaccharide ‘boost’—may be ‘shock’-like cytokine storm	Provide additional nutritional and hydration support for animals before injection Define appropriate humane endpoints Monitor body mass and dehydration, remove faecal plugs
Effects of arthritis	
Painful joints, sore feet, lameness, disability and distress	For intra-articular induction protocols only induce in a single joint Implement husbandry refinements, e.g., weighing boats to sit in, refuges designed so that animals do not have to turn around, long nozzles on drinking bottles, soft sawdust litter, short and soft nesting material (long strands can wrap around sore legs) Provide soft, appetising diet or diet gel (accustom animals to this before the acute phase) Pick up and handle using washed Vetbed^®^ Handle very gently and empathetically Give analgesia if possible (see Sect. 5) Refine humane endpoints; include consideration of study duration and the level of disease severity necessary to answer the scientific question
Acute pain	Provide analgesia if possible, e.g., opioid during ‘attack’ phase Provide appropriate environmental enrichment, and group housing for social animals, to help shift attention from acute pain
Other welfare issues	
Behavioural problems, e.g., aggression	Question scientific justification and necessity for using aggressive strains, or male mice of some strains e.g., DBA1, C57BL/6 Use littermates where possible Review husbandry with respect to group size and number/design of refuges If single housing is necessary for welfare reasons, ensure animals have adequate enrichment, especially a refuge and plenty of nesting material Remove aggressors if necessary
Inherently severe arthritis in particular models, e.g., spontaneous SKG mouse, and species or strains (e.g., Lewis or DA/Ola rat)	Explore potential to answer the same question using a less severe model, e.g., Methylated Bovine Serum Albumin (mBSA) model, or a less susceptible strain

### Housing and care refinements

Husbandry refinements, including appropriate environmental enrichment, benefit animal welfare and should be provided unless there is sound scientific justification to withhold them. However, it is essential to evaluate any effects on data variability (Mikkelsen et al. 2010) and to allow for these within the experimental design.

Based on the human experience of arthritis, affected animals should benefit from being able to keep warm and comfortable, exercise as appropriate and reach food and water easily. Temperature may be especially important for animals in RA studies, since healthy mice, given an opportunity to select their thermal environment, choose an ambient temperature of 30–31 °C, considerably above the range in most facilities (Gaskill et al. 2009, 2012). This suggests that it is good practice to review ambient temperature levels for rodents in RA studies and provide a sufficient quality and quantity of nesting material. Besides the animal welfare implications, the systemic sympathetic response to cold stress can affect data quality in studies relating to immune function (Karp 2012; Kokolus et al. 2013), and it is worth considering how this might also apply to RA projects.

Regarding enrichment, shifting attention away from pain benefits human patients (Ulrich 1984; Chan et al. 2012; Havey et al. 2014), and distraction from pain also modulates pain perception in animals (Gentle and Tilston 1999; Ford et al. 2008). An appropriately stimulating environment will therefore likely help to improve welfare and reduce pain perception in animals on RA studies.

Standard principles for housing, husbandry and care of mice and rats used in RA studies are:

- *Soft litter*, to reduce pain on walking.
- Sufficient *soft, non-tangling nesting material* to keep comfortable, cushion sore joints and enable thermoregulation.
- An appropriate group of *cagemates for social animals*, depending on age, sex and strain.
- One or more *refuges*, to permit natural behaviour and alleviate potential anxiety in animals with compromised mobility.
- Effortless *access to easy-to-eat food and water*, to cater for disability.
- *Appetising food*, to counteract or prevent weight loss.
- *Proactive* welfare management as opposed to *reactive.* For example, animals should be acclimatised to cage provisions, appetising food and hydration agents *before* arthritis is induced.

Defined sources of enrichment items should be used, because contaminants (e.g., dioxin), present in some oils and bleaching agents, can act as confounds (e.g., by affecting Cyp1A1 gene activity; Tischkau and Mukai 2009). Standardisation can be managed in the same way as regulatory toxicology studies, in which in-house Quality Assurance groups set limits of acceptability for different substances in litter, nesting materials and enrichment items. These limits are used to review Certificates of Analysis that accompany such materials. Describing the sources of all materials that are provided will help others to interpret the results and conclusions of publications (Hutchinson et al. 2005).

### Catching animals prior to handling

It has been demonstrated that being caught by the tail is stressful for mice and induces anxiety (Hurst and West 2010; Gouveia and Hurst 2013). Avoiding capture by the tail in RA studies, by catching and restraining using cupped hands, tunnels or Vetbed^®^, could thus decrease stress as well as reducing the risk of causing discomfort through involuntary extension/flexion of arthritic joints, or if animals have been injected close to the tail base.

### Arthritis inducers and their administration

Local adverse effects due to inducers can be minimised by reducing the dose, volume and frequency of administration. The feasibility of this approach depends on the nature of the antigen, its solvent, and the adjuvant, and can be evaluated in pilot titration studies, using scoring systems to monitor the onset and severity of arthritis.

Regarding administration, wide gauge needles cause more pain and increase the risk of ‘tracking’, where a tunnel remains under the skin following withdrawal. The inducer can leak into this, causing irritation and further discomfort or pain. To avoid tracking, withdraw the needle slowly and smoothly, applying slight pressure with the thumb. Mycobacteria for adjuvant and collagen-induced arthritis should be ground very finely to prevent ‘stacking’ in finer needles (Brand et al. 2007) and consequent variability in incidence and response. NB Care must be taken when alternating between strains of *Mycobacterium*, as *M . butericum* can induce more severe rat adjuvant disease than *M. tuberculosis*.

Many protocols use intradermal injection, but this leads to a higher incidence of ulcers, especially if administered close to the tail. In the experience of Group members, subcutaneous injection at single or multiple sites further from the tail (e.g., on the flank) can reduce ulcer incidence while still reliably inducing arthritis. This may be possible in some studies that do not depend on intradermal injection, and a pilot study to evaluate an alternative administration protocol may be justifiable. If there is scientific justification for intradermal injection, sites should be chosen with care, taking into account both the animal’s movements and areas that will be affected during restraint.

### Study duration

Refinement can also be achieved by reducing the duration of experiments, provided this is compatible with the study aims, i.e., all the necessary data can be obtained within the study time. There may also be scientific reasons not to prolong studies, due to the risk that the disease will enter the ‘repair phase’, with associated periostitis or ankylosis that can confound the results.

## Adverse effects of RA models

Some factors that require special attention are set out below.

### Boosting

Boosting is not used in rats, but is sometimes used in mice to support antibody-induced collagen induced arthritis, or to synchronise the collagen-induced arthritis (CIA) model. Boosting commonly takes the form of an intraperitoneal injection of lipopolysaccharide (LPS) or subcutaneous administration of collagen in Freund’s incomplete adjuvant (FIA); Freund’s complete adjuvant (FCA) should not be used due to the challenge response to the adjuvant.

Administering LPS is stressful and induces cytokine release that can cause severe adverse effects including shock, diarrhoea, and malaise. Animals may not drink, and may develop faecal plugs that, coupled with diarrhoea, can be life-threatening if not checked regularly and cleaned away. However, there may be justification for using LPS if it allows study duration to be reduced and/or fewer animals used because the incidence of arthritis is reliably increased. Collagen/FIA boosting should be subcutaneous, and can be refined by restricting sensitisation to one side of the animal and administering the boost at a separate site. The choice of ligand should also take translatability and adverse effects into account; for example, the various TLR ligands may model different aspects of RA more effectively than more commonly used agents such as LPS or FIA, but may have different adverse effects.

### Acute phase of arthritis

Animals experience the most severe pain during the acute or ‘attack’ phase, during which they need close monitoring and additional care, including analgesia if feasible (see Sect. 5). Most therapeutic studies are carried out during this acute phase and it is not usually necessary to extend them into the chronic, resolving phase (see below). The severity of the acute phase should be refined to provide the required statistical power for the primary outcome and not more.

### Resolving phase

If polyarthritis models are taken into the ‘chronic resolving’ or ‘recovery’ phase (e.g., to study bone remodelling), the impact on the animal must be carefully considered. The consequences of periostitis become more prominent (especially in rat adjuvant arthritis) as aberrant bone outgrowths develop within the paws and along the bone shafts. Although acute inflammation may have receded, periostitis and spondyloarthropathies are painful and add significantly to the lifetime severity.

Joint damage can be so severe in polyarthritis models that resolution to normality is impossible. Low acute-load models should be used to investigate factors influencing resolution, as they allow mechanisms in joint resolution to be seen as well as causing less suffering (Montero-Melendez et al. 2011).

### Control groups

In studies of potential therapeutics, animals in control groups that do not receive the candidate therapeutic agent will develop the most severe form of disease and are of special concern. The requirement, and humane endpoints, for controls should be very carefully considered. In some fields, controls can be avoided by using ‘historic’ controls from the literature or the same institution. Unfortunately, in RA studies this is likely to mislead due to variations between institutions, contemporaneous environmental factors, and the protocols used. However, sharing control groups from different, contemporary experiments within the same institution is valid, and should be encouraged, provided they possess sufficient power.

### Aggression

Inappropriate enrichment can cause aggression in male mice (Marashi et al. 2003), and male DBA/1 and C57BL/6 mice, both of which are used for experimental arthritis, are especially prone to aggression. However, the risk can be reduced by establishing groups early, using littermates, ensuring that animals are not subsequently mixed and selecting appropriately designed refuges. For mice, two refuges or dual entry/exit designs can defuse aggression (T Boden pers. comm.), but if it persists, it may be necessary to remove aggressor(s) since fighting may cause stress, injury and infection—which can all influence arthritis development. Aggression between arthritic rats is not expected and indicates a serious welfare issue that should lead to a review of husbandry and experimental protocols.

### Adjuvant arthritis

Adjuvant arthritis models, such as intradermal/subcutaneous administration of CFA to rats, or pristane to rats and mice, can easily lead to severe outcomes. Severity may be reduced by refining the initiation doses, e.g., reducing the dose of pristane for pristane arthritis, without compromising the arthritis outcomes (Malik et al. 2011).

The severity of CFA arthritis in rats can be compounded by systemic disease that, if uncontrolled, includes liver granuloma (which can affect Cyp enzyme activity), skin disease, splenomegaly, ulceration at the injection site, tail lesions, eye disease and disuse of the hind paws leading to dragging (‘sledging’), and ulceration of the hind feet.

Although there are welfare concerns, there may be scientific benefits to consider as part of a harm-benefit assessment. The ‘structural validity’ of CFA-induced arthritis is different to other models (Patel et al. 2010) with cell mediated immune responses predominating, which may benefit the scientific objectives of some studies. In addition, drug responses can be more easily distinguished (Bolon et al. 2011), and when inbred strains are used responses tend to have low variability, with robust incidence and very predictable disease onset. These points mean that numbers do not need to take variability into account, so can be kept lower than for murine models. Therefore, there may be scientific justification for using CFA, but this should be very closely scrutinised and every opportunity taken to reduce suffering; pilot studies may be advisable before using this model for the first time.

Older publications describe the injection of the inducer into one hind paw, inducing the ‘primary response’, a chronic granulomatous reaction. The ‘secondary response’ of inflammation in the contra-lateral paw reflects the systemic arthritis. This protocol is incapacitating, can lead to severe disease, and should not be used.

### Arthritis in genetically altered (GA) strains

Some GA strains require careful monitoring, as the severity of arthritis may be unexpectedly severe or chronic. For example, prostaglandin-D_2_ synthase knockout mice have an exaggerated delayed-type hypersensitivity response, which would be predicted to result in severe disease if expressed in collagen arthritis (Trivedi et al. 2006); indeed, PGD_2_ antagonism exacerbates disease (Maicas et al. 2012). Where exacerbation of disease is expected in a GA line, models or protocols of reduced severity, such as monoarticular or reduced inducer dose, should be used wherever possible.

## Analgesia in models of RA

All arthritis models involve pain and some are in fact used as models of inflammatory pain (Colpaert 1987; Honoré et al. 1999). Some pain can be attributed to the acute inflammatory response, but chronic pain may also develop, with hyperalgesia associated with a variety of neurochemical changes in the spinal cord (Colpaert 1987; Honoré et al. 1999; Christianson et al. 2010; Bas et al. 2012). This is evidenced by the fact that rats and mice continue to self administer analgesics in the resolving phase, even though paw swelling has abated (Colpaert et al. 2001; Wooley et al. 1981). The goal of analgesia in RA studies is to minimise acute and chronic pain without having a significant negative impact on scientific validity.

### Analgesics and scientific validity

Most analgesics affect the immune system in some fashion (Paska et al. 1986; Earl et al. 1994; Dinda et al. 2005; Pulichino et al. 2006), so there can be concern about providing analgesia for fear of introducing a confound. However, there is increasing evidence of effects upon many body systems (including immunity) of unrelieved pain and distress in animals, and how these can influence the experimental outcome (Baumans et al. 1994; Livingston and Chambers 2000; NHMRC 2008; Ren and Dubner 2010). It is also noteworthy that analgesics are rarely excluded from human clinical trials, which are therefore subject to the same confounding influences.

The authors therefore propose that analgesia should be used unless there is sound scientific justification otherwise, e.g., if it can be demonstrated that analgesia would make it difficult to attribute therapeutic effects to the study compound, or if a class of analgesic could affect unpredictably the disease severity in studies using GA animals. For a discussion of decision making regarding pain alleviation, see Carbone (2011).

Pain relief protocols can be designed to minimise potential impact on the scientific objectives. For example, analgesia can be tailored to periods when inflammation is at its peak and likely to be especially painful (Khachigian 2006; McCarthy et al. 2012), with administration in anticipation of (rather than in response to) the pain in order to increase efficacy.

### Potential analgesics for RA studies

When considering whether to provide analgesia, two essential questions are (i) what effects occur at analgesic doses, and (ii) will these necessarily invalidate experimental outcomes? There are a number of reports of the use of analgesics in arthritis models without negative impacts on the experiment; some examples are set out below.

*Gabapentin* has been found to be effective in the attenuation of allodynia during the chronic phase of murine K/BxN arthritis (Christianson et al. 2010) and has also been shown not to interfere with the immune response (Van Loo et al. 2006). Gabapentin, the NSAID ketorolac and the TNF receptor antagonist Etanercept (R) have all been reported as effective during the acute phase, whereas gabapentin alone was effective on allodynia in the chronic phase (Christianson et al. 2010). Since NSAIDs are anti-inflammatory and can have DMARD activity (Seed and Burnet pers. comm.), anti-TNF is antirheumatic, and both are only effective in the acute phase, gabapentin could be used.

*Buprenorphine*, a partial agonist opiate, does not prevent the development of a reliable arthritic response in mice when administered in drinking water (M. Burnet, Synovo, pers. comm., see below), but neither direct comparisons with non-treated mice, nor effects on joint histopathology, have been assessed. Buprenorphine reduced spinal neuronal discharges and reduced allodynia during the acute and chronic phases of mouse CAIA (Bas et al. 2012). However, oral administration of buprenorphine at analgesic doses (2 mg/kg twice daily) in rat SCW arthritis inhibited inflammation and joint erosion (Volker et al. 2000).

*Paracetamol* (acetaminophen) is potentially a suitable analgesic, since it possesses less anti-inflammatory activity than NSAIDs and COX-2 inhibitors. Paracetamol at 50 mg/kg significantly reduced nociceptive evoked and spontaneous spinal discharges in adjuvant arthritis (McQueen et al. 1991). It also reduced inflammatory hyperalgesia without affecting carrageenan inflammation and central hyperalgesia (Bianchi and Panerai 1996).

Therefore, it is possible to provide analgesia in some RA studies, and some institutions apply analgesia routinely. However, further research is needed into suitable analgesic regimes, including evaluations from the time of induction, or specifically the attack and chronic phases, to assess analgesic efficacy and dissociate these from arthritic outcomes. If in doubt, the veterinarian should be consulted, literature searched and pilot studies conducted if necessary.

### Pilot studies

Pilot studies could establish whether analgesia might be provided at one or more stages of an RA study without significantly compromising the science. Although the animals in the full study will benefit if it is shown that pain relief is feasible, pilot studies to evaluate this will cause suffering and should undergo a harm-benefit assessment, with full implementation of the three Rs. Ideally, the pilot study should be designed such that data from it could potentially be incorporated into the main study, to avoid using ‘extra’ animals.

### Administration of analgesics

There are three main options for analgesic administration; parenterally or orally by either gavage or self-administration. There are pros and cons associated with each of these routes (Table 3). For guidance on administering analgesia in nut paste (e.g., Nutella^®^), see Jacobsen et al. (2011) and Abelson et al. (2012). Others have reported providing buprenorphine in the usual diet (Molina-Cimadevila et al. 2014).

**Table 3:** Advantages and disadvantages of parenteral versus self-administration of analgesics in RA studies

Method	Advantages	Disadvantages
Parenteral administration	Can be reasonably certain that required dose has been reliably delivered New slow-release opioid preparations are being developed which may offer prolonged and sustained analgesia without the need for re-dosing	Capture, handling and restraint for administration may be painful and stressful, especially in the attack phase or if animals are in chronic pain Painful injection, risk of infection, risk of reduced disease on repeated administration A ‘standard’ dose may not be appropriate for the individual
Orally by gavage	Can be reasonably certain that required dose has been reliably delivered Does not require injection	As for parenteral administration, plus gavage procedure can be distressing Some physical risk to animal, e.g., misdosing into trachea, damaging oesophagus
Self-administered in water^a^ or diet	No handling or restraint required Involuntary self administration, or can train animals to recognise water with analgesic Animals can dose themselves optimally Cage intake easily measurable	Individual intake unknown Severe pain could reduce ability to access food or water and thus analgesia May be issues with absorption or bioavailability
Self-administered in ‘treat’ food	No handling or restraint required Can accustom animals to taking treats (e.g., jelly, Nutella^®^) so that they will readily self-administer Animals can dose themselves optimally	As for self-administration in water/diet May not readily take treat with analgesic Rate of intake can vary with several factors, e.g., position in group hierarchy

^a^Example regimes; 240 mg paracetamol in 140 mL drinking water, or 1 mg/L buprenorphine in 150 mL water per diem (T Boden; M Burnet, Synovo GmbH, pers. comm.)

## Assessing animal wellbeing, pain, suffering or distress in RA studies

Species and strains vary regarding susceptibility to arthritis and how pain-coping behaviour is expressed. An effective day-to-day welfare assessment system should therefore be tailored to the species, strain and protocol, with input from the researcher(s), animal technologists and the veterinarian (Hawkins et al. 2011; European Commission 2012, 2013). All those responsible for assessing animals should receive adequate training in recognising indicators of suffering associated with each project and in using the relevant recording systems.

Assessment often involves handling, and animals used in RA studies may be in pain, so they should be habituated to empathetic, careful capture and manipulation. For example, mice or rats with swollen paws should never be picked up from cage lids. However carefully it is done, handling animals during the attack phase is still likely to be painful, so a compromise is needed between ensuring adequate assessment and minimising (or even avoiding) catching and handling. Some indicators can be assessed without handling, such as ‘pain faces’ (below). Note that mice and rats are nocturnal and most active during the dark phase, so important behavioural signs may be missed if animals are only observed when it is light.

The monitoring protocol will also depend upon the study phase, e.g., one author’s facility assesses animals three times a day during the acute phase of CAIA following LPS administration. If ‘rescue analgesia’ has been agreed, daily full assessment (involving handling) is necessary to recognise when pain relief is required. Post-acute phase, animals are generally monitored daily with less frequent detailed assessment. An example regime is assessment every other day from days 5 to 25 and twice a week from then, provided the animal’s condition has stabilised. After the acute phase, animals should be weighed and body condition scored on assessment days.

### Suitable welfare indicators for mice and rats in RA studies

The European Commission (EC) Expert Working Group on Severity Assessment guidance on recording clinical observations sets out ‘high level’ categories of observations, which are then broken down into areas to focus on when observing animals, then specific indicators to monitor within each area (European Commission 2012). Table 4 lists indicators from the EC guidance identified by Group members as most relevant to RA studies, categorised according to the EC system.

**Table 4:** Useful indicators for welfare assessment of mice and rats used in RA studies

High level category	Areas to focus on when observing animals	Specific indicators to monitor
Appearance	Body condition	**Weight loss** and/or loss of body condition
	Coat and skin condition	**Ulceration**
		Faecal or urine staining
		Unkempt or greasy coat
		Scabbing, **ulceration, infection** at injection site associated with adjuvant
		Skin tenting (dehydration)
		In adjuvant arthritis, crusting or lesions around eyes, ears, paws, tail; tail ‘ribbing’ (changes in connective tissue) **Paw ulceration**
	Discharge	Ocular discharge
	Other	‘Pain face’, e.g., semi-closed eyes and nose bulge in mice
Body functions	Respiration	Dyspnoea or tachypnoea
	Food/water intake	Reduced
	Body temperature	Decreased temperature, indicated by observing shivering or use of thermography
Environment	Enclosure environment, including any litter, nesting material, enrichment items	Soft faeces or diarrhoea; or lack of faeces (constipation)
		Poor quality nest
		Reduced use of enrichment items such as chew blocks
Behaviours	Social interaction	Change in temperament or responsiveness. Pain can have varying effects e.g., reduced aggression to conspecifics, or increased aggression towards humans
		Isolated or withdrawn from conspecifics
	Posture and mobility	Lethargy
		Reduced wheel running
	Other	**Vocalisation; spontaneous or invoked**
		Sleep disturbance
		Less willing to take treats or to incorporate new material into nest
Procedure-specific indicators	Indentified on the basis of the individual project, its potential adverse effects and expected indicators of these	**Abnormal gait (e.g., ‘sledging’ in severe cases)**
		**Abnormal posture**
		**Paw swelling**
		Clinical indicators e.g., data from von Frey tests, gait analysis apparatus and software, data from imaging joints
		Analgesia self-administration, where applicable
		Serum biomarkers, if available as part of the project, could be used to provide additional information about disease progression—but blood samples should not be taken solely for this purpose
**Free observations**	A severity assessment scheme should always include a facility to note any observations of unexpected indicators of suffering	

Indicators in bold are especially relevant with regard to humane endpoints

The EC has also published a worked example of this approach for Type II CIA in rats (European Commission 2013), and “Appendix” to this document sets out an example hypothetical ‘score sheet’ for mice using some of these indicators. Note that this is a generic example, not suitable for use without adaptation and tailoring to the species, strain and protocol. Some of the indicators and their applications are explained further below.

#### Weight loss

Animals on RA studies may lose weight, or weight may remain stable when an animal should be growing, either of which can be a concern. Scores are generally assigned for percentage weight loss. It is good practice to obtain the baseline weight for each animal and note the weight at which to implement the humane endpoint. In growing animals, it may be necessary to compare with age-matched controls.

Body condition scoring can be used to assess body fat and/or muscle mass loss (Ullman-Culleré and Foltz 1999), but this involves handling and palpating animals which may be painful. Discomfort can be reduced by using a clean piece of Vetbed^®^ to restrain animals, or to rest them on during handling.

#### Running wheels

Reduced levels of running wheel use can infer levels of joint pain (Krug et al. 2009; Whittaker and Howarth 2014), but there is currently debate about running wheels as ‘enrichment’. Some view them as providing additional activity, and in the experience of one author wheels help to reduce aggression. However, there are concerns that wheel running is an abnormal, and possibly addictive, behaviour because animals spend long periods running, sometimes to the detriment of other behaviours (Sherwin 1998; Würbel 2008; Richter et al. 2014). The utility of running wheels as an indicator of RA progression may justify providing them, provided that average group data are obtained rather than singly housing social animals to acquire individual data.

#### ‘Pain faces’

Research is ongoing into ‘pain faces’ in a number of species. A mouse grimace scale (MGS) and rat grimace scale (RGS), including elements such as ‘orbital tightening’ and ‘nose bulge’, have been developed for use when animals are experiencing acute pain (Langford et al. 2010; Sotocinal et al. 2011; Whittaker and Howarth 2014). These Grimace Scales can be useful for assessing animals in chronic pain if this also includes acute episodes. At one author’s establishment the MGS is displayed in procedure rooms to help staff assess animals.

#### Disturbed sleep

Disturbed sleep has been noted in animals on RA studies (Andersen and Tufik 2000). It is unlikely that many facilities will have the resource to monitor sleep patterns at present, but non-invasive biotelemetry systems and behavioural monitoring software are both developing rapidly and may become more accessible. Such systems could also be used to monitor animals when active at night.

#### Swollen paws

**Fig. 1:**
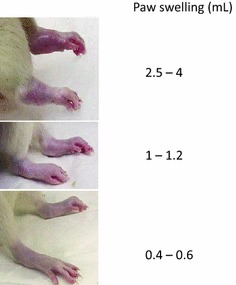
Degrees of paw swelling in FCA adjuvant arthritic rats, measured using plethysmography. This illustrates significant, but well controlled, paw swelling to 2.5 mL. Volumes above this are likely to cause severe pain and debilitation and should be considered a humane end point, as in the *top paw*. (From Bolon et al. (2011), reproduced by kind permission of Hindawi Publications Corp.)

**Fig. 2:**
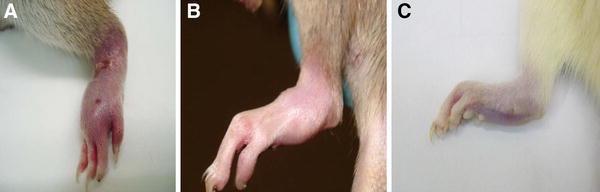
Appearance of rat hind paws with arthritis following different doses of pristane. **a** Note swelling, redness and start of skin lesions. **b** Ankylosis at the chronic phase; histology shows active inflammation. The animals shown in **a** and **b** reached the humane endpoint and were humanely killed. **c** Well managed arthritis. The development of severe arthritis with lesions, as in **a**, is not required as power can be maintained with lower doses of pristane as in **c**, also reducing variability. (Courtesy M. Seed, University of East London)

Animals should be very gently caught and handled, and paws checked daily, from around day 14 following the initial induction (or from the point of inflammation). The digits and joints should be examined, and if there is swelling it should be noted how high up the limb this is present. Swelling may be measured with (preferably non-spring) callipers or by plethysmometry, as swollen paws are painful (Bolon et al. 2011) (Fig. 1). Swollen paws and digits may also be accompanied by reddening of the skin (Fig. 2).

**Fig. 3:**
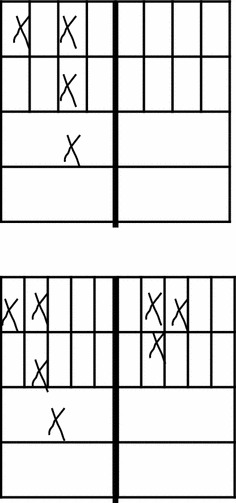
System for recording paw scores. An example of a scoring system that can be used in pilot rodent trials to determine the pattern of disease expression and evaluate different scoring systems and determine power. The *top rows* represent the digits, *second rows* are the knuckles, and *third* and *fourth rows* are the midfoot and ankle/wrist respectively. The scores in this case are: left front 4, right front 0, left hind 4, right hind 3 (courtesy M. Seed, University of East London)

An example approach to visually monitoring clinical scores of the hind paws in pristane adjuvant arthritic rats is outlined below (Table 5; Fig. 3). In this trial, all joints were assessed in order to determine an optimal scoring system relevant to that model, as different joints develop and resolve arthritis at different times.

**Table 5:** Example scoring scheme for investigating the pattern of paw involvement in pristane arthritis, using DA rats to determine outcomes for the full trial. This can be adapted for use with other models

Arthritis score	Maximum points—hindpaws	Maximum points—forepaws
1 point for each swollen or red digit	5	4
1 point for each swollen knuckle	5	4
1 point for swollen midfoot	1	1
1 points for a swollen ankle/wrist	1	1
Total (×2)	24	20

**Fig. 4:**
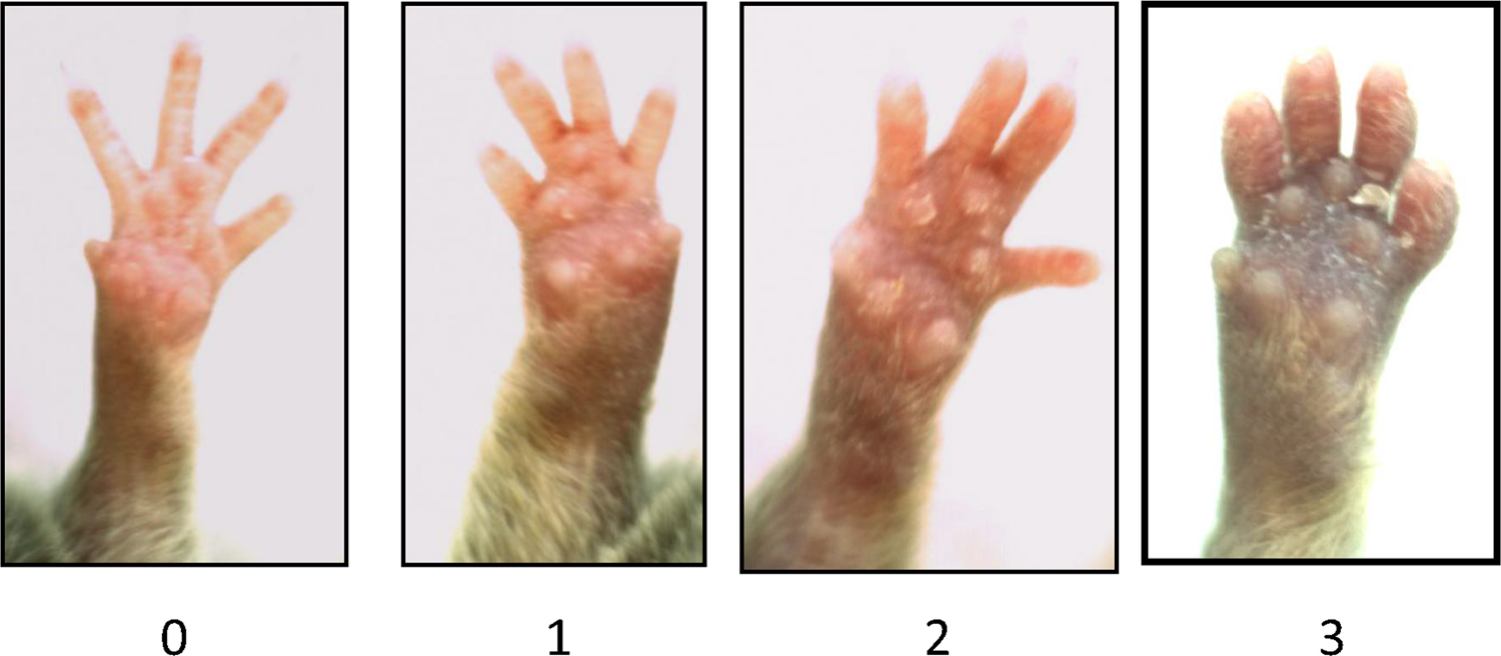
Appearance of mouse front paws with progressively severe CIA arthritis. In this example scheme, *0* normal, *1* digits swollen, *2* digits and pad swollen, *3* wrist/ankle, pad and digits swollen. (Courtesy Remi Okoye, Alex Vugler; UCB Celltech)

In mice, distinguishing individual joints is difficult without handling animals and touching the paws. However, in the authors’ experience an assessment system for mice in which whole paws are scored (Fig. 4) was as robust as a protocol that scored individual digits and joints (see “Appendix”). ‘Global’ scores for each paw, tailored to individual projects, can therefore be used for mice, avoiding handling.

Infrared thermography (using a video camera) has been suggested to monitor clinical severity, as foot temperature can be correlated with the degree of swelling (Jasemian et al. 2011). Thermographic images can be taken without anaesthesia, restraint or otherwise handling the animals, with welfare advantages if joints are painful.

#### Self-administration of analgesics as a welfare indicator

Analgesia self-administration can indicate discomfort or pain (Whittaker and Howarth 2014), either if self-administration is part of the project, or if pain relief can be provided during at least part of the study. It may be justifiable to conduct a pilot study using analgesic self-administration to validate behavioural indicators of pain (Colpaert et al. 1980, 2001), even if analgesia cannot then be provided during the actual study.

## Humane endpoints

‘Humane endpoint’ can be defined as ‘the point at which an animal’s pain and/or distress is terminated, minimised or reduced, by taking actions such as killing the animal humanely, terminating a painful procedure or giving treatment to relieve pain and/or distress’ (see http://www.humane-endpoints.info).

The humane endpoints for each study will depend upon factors including its aims, the stage at which sufficient data are obtained, and sometimes whether a predetermined ‘severity limit’ has been reached. Local ethics or animal care and use committees may also have input into defining humane endpoints. However, the authors believe that there are some generally applicable limits regarding specific adverse effects in RA studies, at which point animals should be humanely killed. These are listed in Table 6.

**Table 6:** Humane endpoints for mice and rats in RA studies

Adverse effect	Humane endpoints and comments
*Ulceration* may develop in induced models, but with good practice this should only occur in a small proportion of mice and rats (<10 % in the authors’ experience)	The focus is generally not on diameter, but on whether there are signs of healing, any secondary infection, ulcer depth, whether the ulcer is wet, and behavioural signs of pain or discomfort However, if an ulcer diameter is >5 mm, the veterinarian or senior animal technologist should be informed and consulted about suitable treatment. The animal should be humanely killed if there are no signs of healing within 3 days
*Abnormal gaits and postures*	‘Sledging’, i.e., pushing the tail down to compensate for two painful hind paws Difficulty holding food Prolonged (>72 h) failure to weight bear on a limb
*Severe paw swelling*, assessed by visual scoring or using callipers or plethysmography (Figs. 1, 2, 4) Paw size increases may need to be corrected for growth	The researcher, animal technologists and veterinarian should collaborate to define the maximum level of swelling, the number of paws that may be affected, how this will be assessed, and how long severe swelling should be permitted to continue Humane endpoints should always be implemented if swelling forces the digits to splay, or encompasses the entire foot and ankle, beginning to rise up the lower leg
**Spontaneous vocalisation** or squeaking and quivering when picked up or handled Arthritic rats can also vocalise in the cage when jostling with cagemates	Rodents generally vocalise at ultrasonic frequencies, so audible calls can indicate severe pain or distress Absence of vocalisation does not indicate acceptable pain levels, as clinical scores can reach humane end points without audible vocalisation
*Weight loss* Exceptions can be made for treatments expected to induce weight loss, such as glucocorticoids	20 % is generally used in RA studies, or some protocols factor in the duration, e.g., endpoint of 15 % loss that does not begin to reverse within 5 days Condition scoring can also be used, with scores that indicate humane endpoints of additional food and/or hydration support or humane killing
*Study-specific end points* These involve an additional pain or anaesthesia burden, so should be used only if data are available as part of the scientific output of the study	Peripheral blood biomarkers may be used as earlier primary indicators of the inflammatory response, rather than gross inflammatory load, e.g., acute phase proteins or urinary cartilage breakdown products might be present before clinical deterioration and suffering Data from imaging joints using X-ray or micro-computer tomography (µCT), e.g., erosion and periostitis endpoints

Other indicators are commonly used in combination to implement humane endpoints, but some involve subjective judgements and using several indicators requires careful consideration. A ‘score sheet’ approach, either noting whether indicators are present/absent or assigning numerical scores to these, can provide a useful adjunct to competent and empathetic human observers by helping to improve objectivity (see Sect. 6).

A number of judgements will need to be made including: the numerical score at which an animal should be humanely killed; whether one or more factors should be weighted; and whether and how duration should be taken into account (e.g., how long to maintain a medium/high scoring, but below threshold, animal). Issues like these should be discussed by the researcher, veterinarian, animal technologists and care staff, with appropriate input from the regulator and ethics or animal care and use committee.

## Experimental design and reduction

Good experimental design is critical with respect to ensuring that projects are statistically robust, and that the correct number of animals is used to achieve the experimental objectives—neither too many, which causes avoidable suffering; nor too few, which is unethical if it means that animals are used in projects that have no significant benefit. For generic guidelines and principles to apply at the project planning stage, see Festing et al. (2002), Bate and Clark (2014) and the ARRIVE guidelines on reporting animal use (Kilkenny et al. 2010). Although ARRIVE primarily relates to writing up in vivo research, it is also a useful study design checklist and is available within the UK National Centre for the three Rs (NC3Rs) resource hub on experimental design; see http://www.nc3rs.org.uk/experimental-design.

In practice there are different, and sometimes competing, factors to take into account when determining appropriate numbers, including the experience of each individual animal. For example, it is sometimes necessary to balance welfare against group sizes, but the authors believe that welfare considerations should predominate. That is, it can be preferable in principle to use more animals, with less suffering to each individual (or better welfare), provided that scientific integrity is not compromised.

## Reporting animal use in RA studies

Literature reviews have identified serious issues with the design, analysis and reporting of animal use in a significant number of publications (Kilkenny et al. 2009; Baker et al. 2014; Bara and Joffe 2014; Moja et al. 2014). The authors support the ARRIVE guidelines and believe that it is critically important to include information on efforts made to replace animals, reduce their use and suffering and improve their welfare in materials and methods sections, or as supplementary materials depending on the journal’s approach and policy (Osborne et al. 2009). Posters and talks can also include brief information on the three Rs, or supplementary information can be included on flyers to accompany poster presentations. This will help to disseminate good practice and enable proper interpretation of the results.

## Recommendations for the future of RA research using animals

Future work should aim to further reduce lifetime severity in RA studies, whilst maintaining or even improving opportunities for medical advances. This would be achieved through the following:

- Further research into the use of analgesia, with respect to suitable agents, effects on welfare and the science, timing of administration and self-administration.
- A systematic review of the provision of analgesia in arthritis experiments.
- Improved indicators of pain and distress, such as accessible computer-assisted behavioural analysis.
- Less severe models, e.g., not requiring the use of potentially severe inducers such as CFA.
- More physiologically relevant spontaneous models using GA mice, which will decrease the number of procedures because it will not be necessary to induce RA.
- International guidelines for refined experimental protocols, including humane endpoints.
- Better sharing and publication of all three Rs in RA studies.
- Greater support for the development and uptake of in vitro, in silico and epidemiological approaches to RA research.

## Conclusion

The Working Group believes that there is considerable scope to reduce the suffering and improve the welfare of mice and rats in RA studies, and hopes that this resource will support and encourage ongoing efforts towards this important goal. Table 7 sets out some key recommendations taken from the text, which can be used as a check list when designing, conducting or reviewing projects, to help reduce severity and ensure that appropriate refinements have been implemented wherever possible.

**Table 7:** List of key recommendations to help refine the use of mice and rats in RA studies

Recommendation	Sections
Review the sources of variation listed in Table 1 and ensure that each is addressed	2.3
Set out the whole life experience of each animal and consider how each potentially painful or distressing event could be refined, using Table 2	3
Use the list of principles in Sect. 3 to review housing and husbandry, addressing any omissions	3
Critically question any statements that environmental enrichment has a negative impact on data quality; ask for empirical evidence and be prepared to conduct or permit pilot studies if appropriate	3
If using an inducer, review its nature, formulation and administration protocol	3
If using LPS or CFA, review the justification and necessity and ensure appropriate refinements and humane endpoints	4
Review criteria for humane endpoints regarding ‘maximum end point responses’ and ‘therapeutic dosing regimes’. If responsible for designing projects, suggest the topic for discussion by the ethics or animal care and use committee	4
For studies within the chronic resolving phase, critically review the model, the potential to reduce acute phase severity and duration, welfare assessment protocols and humane endpoints	4
Review the justification and necessity for control groups, sharing these wherever possible without compromising the science, and refining humane endpoints	4, 7
Do not assume that analgesia will negatively affect data. Use the literature, and undertake or permit pilot studies as necessary, to evaluate the effects of analgesia on both welfare and science	5
Carefully consider how to administer analgesia, including pros and cons of gavage, parenteral- and self-administration	5.4
Keep up with developments in animal monitoring technology, e.g., new software, activity meters and thermography—avoiding those that require single housing	6
Ensure that appropriate welfare assessment protocols are defined, and regularly reviewed, with a variety of inputs including the veterinarian, researchers, animal technologists and the ethics or animal care and use committee	6
Implement the ‘R’ of reduction thoughtfully, ensuring that sufficient power is maintained while minimising numbers and severity	8
Use the ARRIVE guidelines as a checklist when designing projects as well as when writing papers for publication	9

## Figures and Tables

**Table 8:**
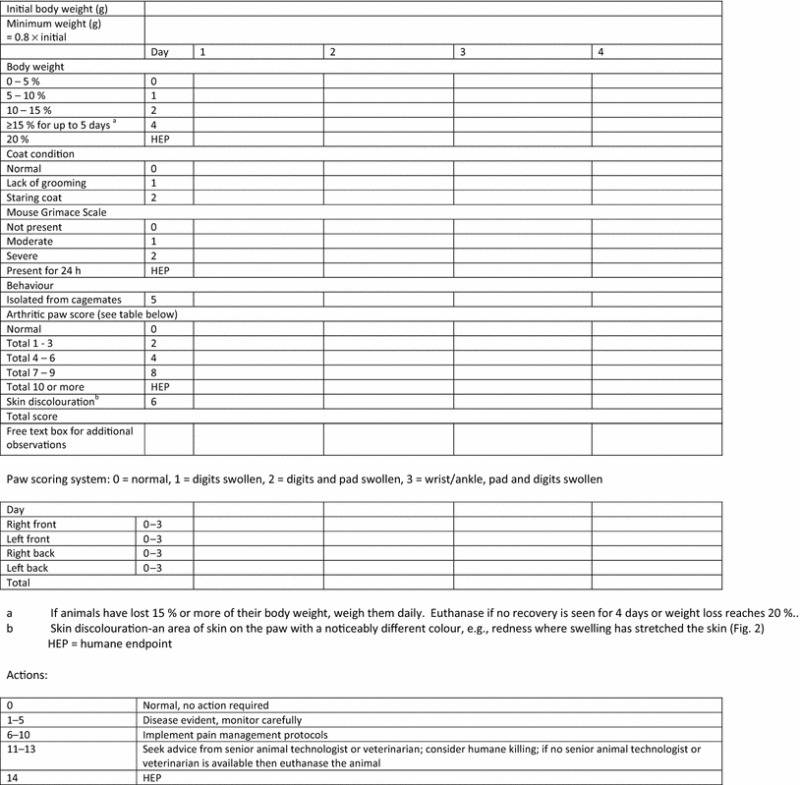
Generic example of a score sheet for mice used in rheumatoid arthritis studies

This should be tailored to meet the characteristics and requirements of individual projects

## Appendix

See Table 8.

## Footnotes

1 Note that in vitro work can also raise ethical and welfare issues, e.g.,, if primary cell lines are generated or animal serum used for cell culture.
